# Incidence and time trends of brain metastases admissions among breast cancer patients in Sweden

**DOI:** 10.1038/bjc.2012.163

**Published:** 2012-04-24

**Authors:** G Frisk, T Svensson, L M Bäcklund, E Lidbrink, P Blomqvist, K E Smedby

**Affiliations:** 1 Department of Medicine Solna, Clinical Epidemiology Unit, Karolinska Institute Solna, Stockholm 171 76, Sweden; 2 Department of Medicine Solna, Unit for Experimental Cardiovascular Research, Karolinska Institute Solna, Stockholm 171 76, Sweden; 3 Department of Oncology-Pathology, Karolinska Institute, Karolinska University Hospital Solna, Stockholm 171 76, Sweden

**Keywords:** breast cancer, brain metastases, incidence

## Abstract

**Background:**

While treatment for breast cancer has been refined and overall survival has improved, there is concern that the incidence of brain metastases has increased.

**Methods:**

We identified patients in Sweden with incident breast cancer 1998–2006 in the National Cancer Register, and matched these to the National Patient Register to obtain information on hospital admissions for distant metastases. Hazard ratios (HRs) and 95% confidence intervals (CIs) were computed with Cox regression as estimates of relative risk.

**Results:**

Among 50 528 breast cancer patients, 696 (1.4%) were admitted with brain metastases during median 3.5 years of follow-up. Admissions for other metastases were found in 3470 (6.9%) patients. Compared with the period 1998–2000, patients diagnosed with breast cancer 2004–2006 were at a 44% increased risk of being admitted with brain metastases (HR 1.44, 95% CI 1.13–1.85).

**Conclusion:**

The incidence of admissions with brain metastases in breast cancer patients was increasing in the mid-2000s in Sweden. These findings support a true increase in incidence of brain metastases among breast cancer patients.

The occurrence of brain metastases in breast cancer patients is believed to have increased (Lin and Winer, 2007), but available supportive evidence is scarce (Smedby *et al*, 2009). If so, this may be an effect of increased incidence of primary breast cancer (National Board of Health and Welfare, 2011b), technical advances in neuroimaging, or a true increase in incidence due to modifications of systemic treatment schedules (Barnholtz-Sloan *et al*, 2004; Gavrilovic and Posner, 2005) and improved survival. Adjuvant and systemic therapy with low penetrance through the blood–brain barrier, such as trastuzumab, may, while decreasing risk of distant metastases in general and prolonging overall survival, lead to an increase in risk of brain metastases in breast cancer patients (Tomasello *et al*, 2010).

We recently showed that the number of admissions to hospital of patients with brain metastases in general, and especially due to breast cancer, increased in Sweden from 1987 to 2006, but we could not account for a parallel increase in primary breast cancer incidence nor for temporal changes in admission practices (Smedby *et al*, 2009). In the present study, we used nationwide health-care registers to investigate time trends in incidence of admissions for brain metastases and other distant metastases among patients with breast cancer during the period 1998–2006, with uniform classification of admission records.

## Materials and methods

We identified 58 795 patients with breast cancer in the Swedish National Cancer Register (NCR) from 1 January 1998 to 31 December 2006. Previous cancer patients were excluded, leaving 50 528 patients in the cohort. The NCR is estimated to be >95% complete (Barlow *et al*, 2009). The cohort was then matched to the National Patient Register, NPR, (National Board of Health and Welfare, 2011a) by the national registration numbers assigned to all citizens, to obtain information on all hospital admissions for distant metastases during follow-up. The NPR contains individualised information on ICD diagnoses associated with all hospitalisations nationwide since 1987, with an estimated proportion of register drop-outs of <2%. TNM classification data from the primary breast cancer diagnosis were available from 2002 (no restrictions were made based on TNM stages). The cohort was also matched to the National Cause of Death Register (National Board of Health and Welfare, 2011c). The study was approved by the Stockholm Regional Research Ethics Board.

### Statistical analyses

The number of incident cases of breast cancer and proportion admitted with brain metastases and/or other distant metastases are presented and compared per time periods 1998–2000, 2001–2003, and 2004–2006. Only the first admission with brain metastases or with metastases at any site outside of the brain (‘other distant metastases’) was considered. Patients recorded with brain metastases as the only metastatic site at their first admission were denoted as ‘First brain metastases’. For patients admitted with brain metastases at the same time, or subsequent to admissions for other distant metastases, we used the term ‘Later brain metastases’. We assessed the 1-, 2-, and 3-year cumulative incidence of brain metastases admissions by year of primary diagnosis, and the incidence was plotted graphically with the Kaplan–Meier method by 3-year calendar periods. We used a multivariate Cox proportional hazards model adjusted for year of birth to compute hazard ratios (HRs) and 95% confidence intervals (CIs) as a measure of the relative risk of admissions for distant metastases in the brain or other sites comparing 3-year periods. *No direct statistical comparisons were made between risk of admissions for brain metastases and other distant metastases.* All individuals were followed from the date of primary breast cancer to date of death, or 31 December 2006, whichever came first. The patients were censored if they contracted another cancer during this time.

## Results

In the cohort of 50 528 patients with breast cancer, 696 (1.4%) were admitted with brain metastases during a median follow-up of 3.5 years (Table 1). Three-hundred and thirty-six (0.7%) patients were admitted with brain metastases as their first distant metastasis and the remaining 360 (0.7%) were admitted for brain metastases in parallel with or subsequent to metastases at other sites. Admissions with other distant metastases were found in 3470 (6.9%) patients. The majority of the patients (97%) diagnosed from 2002 and onwards did not have clinically evident systemic disease (M1) at diagnosis.

The median time between diagnosis and first admission for brain metastases was 2.3 (1.3–4.0) years and shorter for those with a first metastasis to the brain (1.8 years) than those who were admitted with brain metastases as part of a widespread disease (2.9 years) (Table 1). The prevalence of survivorship at the end of each 3-year period of primary diagnoses increased marginally from 93.0% 1998–2000 (*n*=15 038) to 93.9% 2001–2003 (*n*=16 060) and 93.8% 2004–2006 (*n*=16 184). Survival among patients admitted with brain metastases was median 3.1 years from primary breast cancer diagnosis, and 3 months from the day of first admission for brain metastasis (Table 1).

**Figure 1:**
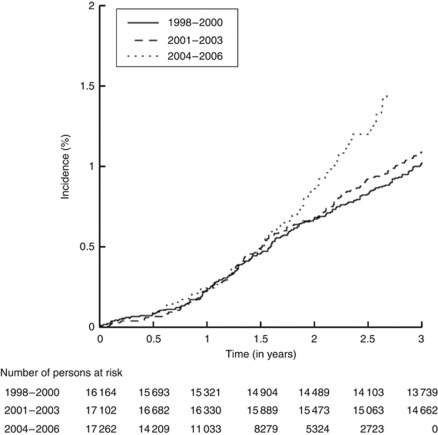
Cumulative incidence of admissions for brain metastases in patients with breast cancer from 1998 to 2006 in Sweden.

The incidence of admissions with brain metastasis during the first year after breast cancer diagnosis varied from 1.1 to 3.1 per 1000 person-years, from 2.7 to 4.4 per 1000 person-years during the first 2 years of follow-up, and from 2.7 to 4.2 per 1000 person-years (Supplementary Table 1). Rates were lowest in 1999 and increased through 2004–2005. Compared with the period 1998–2000, patients diagnosed with a primary breast cancer during 2001–2003 were at 17% increased risk of being admitted with brain metastases (HR 1.17, 95% CI 0.99–1.39) during follow-up, and patients diagnosed 2004–2006 were at a 44% increased risk (HR 1.44, 95% CI 1.13–1.85) (Figure 1). The risk was more pronounced for later brain metastases (HR 1.80, 95% CI 1.23–2.63) than for first brain metastases (HR 1.21, 95% CI 0.87–1.68) in 2004–2006, compared with the period 1998–2000. The relative risk of admissions with other distant metastases was 1.02 (95% CI 0.95–1.11) 2001–2003 and 1.11 (95% CI 1.00–1.24) 2004–2006, compared with the period 1998–2000. When assessing the risk of brain metastases by time of follow-up, the increased risk was primarily observed 1.5 years and onwards after primary breast cancer diagnosis 2004–2006 (HR 2.07, 95% CI 1.45–2.94) than during the first 1.5 years (HR 1.08, 95% CI 1.76–1.54), compared with the period 1998–2000 (Figure 1).

## Discussion

In this large population-based register study of patients with incident breast cancer, we observed a 44% increase in risk of being admitted with brain metastases among patients diagnosed 2004–2006 compared with the period 1998–2000. Risk of admissions for other distant metastases did not increase to the same extent. Our findings further indicated that the rise in incidence of brain metastases primarily occurred among patients with widespread metastatic disease, and at least 1.5 years after the primary breast cancer diagnosis. Although the study is limited to admissions with brain metastases, our findings support a true increase in incidence of brain metastases during this time.

Two previous studies found no evidence for an increase in the incidence of brain metastases in breast cancer (Schouten *et al*, 2002) or in patients treated with trastuzumab (Lai *et al*, 2004). However, both studies spanned earlier time periods. In a more recent study, Pelletier *et al* (2008) observed an increase in incidence of brain metastases in breast cancer patients from 6.61% in 2002 to 10.92% in 2004. The cumulative incidence of brain metastases admissions in our cohort was 1.4%, which is a low incidence estimate compared with previous studies (Schouten *et al*, 2002; Barnholtz-Sloan *et al*, 2004; Gavrilovic and Posner, 2005; Tomasello *et al*, 2010). Our estimate based on hospital admissions is however bound to primarily represent individuals with severe symptoms requiring inpatient care rather than patients with subclinical disease. Also, median follow-up was shorter in our study than in other studies (Schouten *et al*, 2002; Barnholtz-Sloan *et al*, 2004). The patients who developed brain metastases were younger at primary breast cancer diagnosis than in the cohort overall, corresponding with the findings of others (Lin and Winer, 2007; Tomasello *et al*, 2010). The median survival from first admission with brain metastases was only a few months, also in line with other studies (Lin and Winer, 2007; Tomasello *et al*, 2010).

Breast cancer characteristics associated with more aggressive disease, ER and PR negativity and HER-2 overexpressing tumours (Gutierrez and Schiff, 2011; Stuckey, 2011) have been associated with an increased risk of brain metastases (Lin and Winer, 2007; Tomasello *et al*, 2010). Adjuvant anti-HER-2 antibody treatment, trastuzumab was introduced in 2000 and was gradually adopted in Sweden. It has been suggested that the introduction of trastuzumab may have altered the natural history of patients with HER-2-positive tumours and unmasked the CNS as a potential tumour cell sanctuary (Lin and Winer, 2007). Alternatively, an increase in incidence of brain metastases may be the result of more efficient palliative treatment and an improved survival among patients with metastatic disease. In a recent Swedish regional study, Foukakis *et al* (2011) reported an improved survival among patients with metastatic disease 60 years or younger for the period 2000–2004, but not among older patients.

The present population-based register study features near complete coverage of primary breast cancer patients and hospitalisations in Sweden and virtually no loss to follow-up. We cannot exclude that our findings are due to more frequent use of advanced radiological techniques such as CT and MR, which may lead to the detection of more cases of brain metastases. However, in our previous study of brain metastases among all cancer patients, admissions did not occur more frequently over time among patients with cancer types other than in the breast and lung (Smedby *et al*, 2009). Temporal changes in how we register disease at admission could also have influenced our results. However, risk of admissions for other distant metastases did not increase to the same extent in the present study. In addition, the number of hospital beds has decreased in Sweden during the last decade (OECD Health Data, 2005). *Hence*, *the observed increasing trend cannot be due to facilitation of hospital admissions over time*. A limitation of our study was the inability to account for stage at primary breast cancer diagnosis. However, since population screening with mammography has resulted in a relative increase of patients diagnosed with early stage breast cancer (Shen *et al*, 2011), our results cannot feasibly be ascribed to an increased incidence of breast cancer patients with stage IV.

To conclude, we found that the incidence of admissions with brain metastases among patients with breast cancer has increased over time in Sweden. If this also holds true for incidence of brain metastases *per se* needs to be confirmed in other population-based investigations with direct ascertainment of recurrence in the brain, and with access to more detailed data on breast cancer characteristics.

## Figures and Tables

**Table 1:** Characteristics of patients diagnosed with breast cancer in Sweden 1998–2006, and number and proportion of patients admitted to hospital with distant metastatic disease in the brain or other sites^a^

			**All brain metastases**	**Other distant metastases**	**First brain metastases**	**Later brain metastases**
	*n* (%)	Follow-up median (IQR) years	*n* (%)	*n* (%)	*n* (%)	*n* (%)
All patients	50 528	3.5 (1.6, 5.8)	696 (1.4)	3470 (6.9)	336 (0.7)	360 (0.7)
1998–2000	16 164 (32)	6.8 (5.4, 7.8)	327 (2.0)	1698 (10.5)	154 (1.0)	173 (1.1)
2001–2003	17 102 (34)	4.1 (3.3, 5.0)	267 (1.6)	1254 (7.3)	124 (0.7)	143 (0.8)
2004–2006	17 262 (34)	1.4 (0.7, 2.2)	102 (0.6)^b^	518 (3.0)^b^	58 (0.3)^b^	44 (0.3)^b^
Age, median (IQR)	61 (52, 73)		53 (45, 63)	61 (51, 74)	55 (46, 64)	53 (44, 61)
Time from breast cancer diagnosis to admission for first distant metastasis, median (IQR) years			2.3 (1.3, 4.0)	2.9 (1.6, 4.8)	1.8 (1.2, 3.3)	2.9 (1.7, 4.5)
Survival from breast cancer diagnosis, median (IQR) years			3.1 (1.7, 4.9)	3.3 (1.7, 5.6)	2.6 (1.5, 4.6)	3.4 (2.1, 5.1)
Survival from first brain metastasis admission, median (IQR) years			0.3 (0.1, 0.7)	0.5 (0.1, 1.6)	0.4 (0.1, 1.0)	0.2 (<0.1, 0.5)

Abbreviation: IQR=interquartile range.

a Brain metastases admissions were categorised as first brain metastases if this was the only site of metastatic disease at first admission, or as later brain metastases if they were recorded in parallel with or subsequent to metastases at other sites. The ICD-10 codes used were C50.9 (breast cancer), C79.3 (brain metastases), C78 and C79 (other distant metastases). Loco regional metastases were not included.

b Proportions are lower during 2004–2006 due to shorter follow-up of patients diagnosed during this period compared with earlier periods.
