# Supplementary material for: Incidence and time trends of brain metastases admissions among breast cancer patients in Sweden
Source: Br J Cancer. 2012 Apr 24;106(11):1850–3. doi: 10.1038/bjc.2012.163 (PMC3364124; doi:10.1038/bjc.2012.163)
Supplement: Supplementary Table 1 [file bjc2012163x1.doc]

**Supplementary Table. Absolute risk and rate of, and latency to, admissions for brain metastases in patients diagnosed with breast cancer, during one, two and three years following breast cancer diagnosis, by year of diagnosis**

| Year of breast  cancer  diagnosis | N with breast cancer | **One year** | | | | | **Two years** | | | | | **Three years** | | | | |
| --- | --- | --- | --- | --- | --- | --- | --- | --- | --- | --- | --- | --- | --- | --- | --- | --- |
|  |  | N with brain met | (%) | Median time from breast cancer to brain met (years, IQR)* | person years | N/1000 pyr** | N with brain met | (%) | Median time from breast cancer to brain met (years, IQR)* | person years | N/1000 pyr** | N with brain met | (%) | Median time from breast cancer to brain met (years, IQR)* | person years | N/1000 pyr** |
| 1998 | 5 308 | 16 | (0.3) | 0.33 (0.13, 0.82) | 5 145 | 3.1 | 37 | (0.7) | 1.26 (0.51, 1.58) | 10 021 | 3.7 | 61 | (1.1) | 1.60 (0.98, 2.32) | 14 642 | 4.2 |
| 1999 | 5 427 | 6 | (0.1) | 0.56 (0.03, 0.84) | 5 266 | 1.1 | 28 | (0.5) | 1.29 (1.04, 1.66) | 10 264 | 2.7 | 41 | (0.8) | 1.61 (1.25, 2.15) | 14 978 | 2.7 |
| 2000 | 5 429 | 15 | (0.3) | 0.82 (0.61, 0.95) | 5 282 | 2.8 | 37 | (0.7) | 1.08 (0.90, 1.52) | 10 315 | 3.6 | 49 | (0.9) | 1.29 (0.96, 1.86) | 15 093 | 3.2 |
| 2001 | 5 555 | 14 | (0.3) | 0.76 (0.62, 0.95) | 5 424 | 2.6 | 33 | (0.6) | 1.08 (0.88, 1.40) | 10 585 | 3.1 | 54 | (1.0) | 1.53 (1.00, 2.18) | 15 467 | 3.5 |
| 2002 | 5 645 | 11 | (0.2) | 0.70 (0.42, 0.83) | 5 511 | 2.0 | 37 | (0.7) | 1.27 (0.90, 1.48) | 10 761 | 3.4 | 55 | (1.0) | 1.48 (1.19, 2.18) | 15 740 | 3.5 |
| 2003 | 5 902 | 13 | (0.2) | 0.64 (0.42, 0.93) | 5 756 | 2.3 | 40 | (0.7) | 1.19 (0.94, 1.53) | 11 237 | 3.6 | 64 | (1.1) | 1.56 (1.10, 2.45) | 16 448 | 3.9 |
| 2004 | 5 885 | 13 | (0.2) | 0.60 (0.37, 0.80) | 5 722 | 2.3 | 49 | (0.8) | 1.46 (0.98, 1.68) | 11 177 | 4.4 |  |  |  |  |  |
| 2005 | 5 742 | 17 | (0.3) | 0.62 (0.31, 0.84) | 5 587 | 3.0 |  |  |  |  |  |  |  |  |  |  |
| 2006 | 5 635 |  |  |  |  |  |  |  |  |  |  |  |  |  |  |  |

** pyr = person years
